# Biopolymer Fibers of High Strength and Enhanced Orientation by the Synergy of High/Low Molecular Weight Chitosans in Hybrid Biomaterials Processed by Gel Spinning

**DOI:** 10.3390/jfb16110405

**Published:** 2025-10-29

**Authors:** Tuan Anh Tran, Ingo Doench, Arnaud Kamdem Tamo, Shaghayegh Jahangir, Sofia Marquez-Bravo, Pamela Molina, Martin Helmstaedter, Aliuska Morales Helguera, Christian Gorzelanny, Anayancy Osorio-Madrazo

**Affiliations:** 1 Laboratory of Organ Printing, University of Bayreuth, 95447 Bayreuth, Germany; tuan.anh-tran@uni-bayreuth.de (T.A.T.); ingo.doench@uni-bayreuth.de (I.D.); arnaud.kamdem-tamo@uni-bayreuth.de (A.K.T.); shaghayegh.jahangir@uni-bayreuth.de (S.J.); 2 Laboratory for Bioinspired Materials for Biomedical Engineering BMBT, Department of Microsystems Engineering IMTEK, University of Freiburg, 79110 Freiburg, Germany; sofigmb@gmail.com (S.M.-B.); pamela.molina@email.uni-freiburg.de (P.M.); 3 Experimental Renal and Cardiovascular Research, Department of Nephropathology, Institute of Pathology and Department of Cardiology, Friedrich Alexander University of Erlangen-Nürnberg (FAU), 91054 Erlangen, Germany; 4 Department of Nephrology, University Hospital Freiburg, University of Freiburg, 79106 Freiburg, Germany; martin.helmstaedter@uniklinik-freiburg.de; 5 Center of Chemical Bioactives, University of Las Villas, Santa Clara 54838, Cuba; aliuska.morales@gmail.com; 6 Department of Dermatology and Venerology, University Medical Center Hamburg-Eppendorf, 20246 Hamburg, Germany; c.gorzelanny@uke.de; 7 Polymer Materials Engineering IMP, CNRS UMR 5223, University Claude Bernard Lyon 1, INSA Lyon, University Jean Monnet, 69622 Villeurbanne, France

**Keywords:** anisotropic biomaterials, functional composite fibers, bionanocomposites, polymer crystallization, chitosan of low and high molecular weight, gel spinning

## Abstract

High-performance spun bionanocomposite fibers, composed of high-molecular-weight chitosan (HMW), low-molecular-weight chitosan “oligomers” (LMW), and cellulose nanofibers (CNFs), were successfully fabricated via gel spinning of viscous aqueous chitosan (CHI) based formulations into a NaOH coagulation bath. The X-ray diffraction (XRD) analysis revealed that the incorporation of cellulose nanofibers contributed to enhance crystallinity of chitosan in spun fibers. The spinning process, which comprised sequential acidic solubilization, basic neutralization, stretching, and drying steps, produced chitosan/CNF composite fibers with high crystallinity, further enhanced by the incorporation of low molecular weight chitosan. The cellulose nanofibers seem to promote CHI crystallization, by acting as nucleation sites for the nucleation and growth of chitosan crystals, with those latter of LMW further enhancing crystallization and orientation due to higher mobility of shorter polymer chains. Two-dimensional XRD patterns demonstrated the preferential alignment of both CNFs and chitosan crystals along the fiber axis. Increasing the proportion of short-chain chitosan led to a reduction of the viscosity of collodion, facilitating the spinning of solutions with higher polymer concentrations. The X-ray diffraction (XRD) analysis revealed that the addition of low-molecular-weight chitosan (LMW), with an intermediate molecular weight Mw of ~4.4 × 10^4^ g/mol, produced the most significant improvements in the crystallinity index (CrI) and orientation. This structural enhancement corresponded to superior mechanical properties like Young’s modulus, yield stress σ_y_, and stress-at-break σ_b_ of the processed composite fibers. By incorporating that intermediate molecular weight chitosan, a Young’s modulus as high as 20 GPa was achieved for the spun composite fibers, which was twice higher than the modulus of around 10 GPa obtained by adding the lowest molecular weight chitosan of Mw ~ 2.9 × 10^4^ g/mol in the composite, and largely above the modulus of around 5 GPa obtained for fiber just spun with chitosan without incorporation of cellulose nanofibers.

## 1. Introduction

In the field of biomaterials, there is an increasing interest in using bio-based materials, especially biopolymers with relevant biological properties, for the most varied applications in biomedicine [1,2,3,4]. Chitosan (CHI) is a linear polysaccharide composed of a copolymeric structure, consisting of (1→4) linked units of 2-acetamido-2-deoxy-β-D-glucopyranose and 2-amino-2-deoxy-β-D-glucopyranose [5,6,7]. It is primarily derived through the deacetylation of chitin [4]. CHI is regarded as the second most abundant natural polysaccharide, following cellulose [8,9,10,11], and it is mainly extracted from crustacean shells and cephalopod endoskeletons [12,13,14]. Recent interest in chitosan has surged within the fields of bioengineering, biofabrication, and biomedicine [1,15,16,17,18], owing to its favorable biological characteristics, including bioactivity [2], biocompatibility, biodegradability, low toxicity, and hemostatic properties [4,13,19]. The mechanical properties of chitosan are largely influenced by its macromolecular structure and the degree of deacetylation (DA), because both characteristics govern the polymer chains interactions and network formation. Higher molecular weight and longer chains increase entanglement and tensile strength, while a higher DA introduces more free amine groups, enhancing possibility for hydrogen bonding and intermolecular interactions. Together, these structural features determine the stiffness, flexibility, and overall mechanical performance of chitosan-based materials [20,21,22,23,24]. Additionally, its solubility in acidic aqueous environments makes CHI suitable for various processing techniques, including wet spinning [23,25,26]. Wet-spun chitosan fibers have been previously produced using either multifilament spinning, which involves washing the fibers in organic solvents to prevent them from adhering to each other in the yarn [27], or monofilament spinning, which eliminates this issue [22,28,29]. In previous studies, yarns made from multifilament spun fibers have been washed with solvents such as acetone or methanol [30]. Additionally, chitosan solutions for wet spinning have been formulated with organic cosolvents, including methanol [31], isopropyl alcohol [32], and propane-1,2-diol [33].

Cellulose nanofibrils (CNFs), known for their exceptional mechanical properties due to their high crystallinity and aspect ratio, are considered promising green nanoreinforcement agents, also due to their ability to form both intramolecular and intermolecular networks through hydrogen bonding [34]. These properties, coupled with their biocompatibility [1], possible biodegradation [17,35], and the fact that CNFs constitute sustainable materials that originate from plant sources highly abundant in Earth [14,36,37,38,39], make CNFs a promising bio-based reinforcement material for composites in biomaterials for biomedical applications [40,41]. The reinforcement of polysaccharide hydrogels and fibers with nanocellulose has been previously reported [42,43,44,45,46], as well as in film materials produced through a straightforward casting-evaporation technique [47,48]. In the case of wet spinning, it necessitates precise management of solution viscosity and efficient material flow through spinneret orifices to prevent clogging [49]. Furthermore, during the wet-spinning process, uniaxial alignment of both chitosan polymer chains and nanoparticles is anticipated to occur during extrusion, drawing, and drying stages [29,50,51].

Low molecular weight chitosan (LMW) might be another potential reinforcing material in biomedical chitosan fibers. Low-molecular-weight (LMW) chitosan demonstrates higher solubility and lower viscosity, which enhances its bioavailability and facilitates its incorporation into biological systems [27]. The reduced size of LMW chitosan allows for more efficient cellular uptake and interaction with biomolecules, conferring superior antimicrobial, antioxidant, anti-inflammatory, and immunomodulatory properties in comparison to its high-molecular-weight counterpart [9,10]. This makes LMW chitosan particularly advantageous for applications driven by biological activity, including drug delivery, wound healing, and nutraceutical formulations. Polymeric chitosan and chitosan oligomers have been demonstrated to promote the formation of phytoalexins or antimicrobial chemicals, which help restrict disease transmission, owing to chitosan’s strong antibacterial capabilities [52]. Kuyama et al. described the chemical synthesis of various sizes of chitosan oligomers, which resulted in particular biological activity [53]. Lower molecular weight chitosan variations exhibit higher crystallinity, which is a key element in chitosan crystallization during optimization of wet spinning process. Moreover, the biological activity of chitosan is strongly regulated by its molecular weight, degree of deacetylation (DA), solution pH, and other variables, as well as the target organism [54,55]. In biomedical settings, LMW chitosan is notably efficient for anticancer activity [56], oral insulin administration [57], treatment of gastric ulcers [58], liver protection [59], blood cholesterol reduction [60], diabetic control [61], and antioxidant benefits [62,63].

In previous studies performed in our groups, chitosan-based fibers were shown to be cytocompatible and demonstrated antibacterial activity [64,65]. In vivo studies also reported low inflammatory response and slow degradation rate after subcutaneous implantation in rats [65,66]. More specifically, our wet spun chitosan fibers are formulated with low molecular weight chitosan (LMW) of low degree of acetylation (DA = 2.5%), which might bring additional biological activities. The impact of molecular weight (Mw) on the antibacterial activity was recently reviewed and modeled [67]. The intrinsic impact of weight-average Mw can be described as a linear relationship with the antibacterial activity (evaluated as Log(1/MIC), where MIC is the minimum inhibitory concentration when Mw is below a critical molar mass concentration (CMW), with a plateau activity achieved when Mw > CMW. Thus, LMW chitosan, as proposed in the present work, should contribute to a maximum bacterial inhibition effect, combined with improved bioavailability, due to better solubility and faster release of shorter chains from processed fibers. Such bio-availability of LMW chitosan also could improve their anti-oxidant properties [68,69,70,71], and increase cellular intake [72]. Finally, the association of low and high molecular chitosan in wet-spun fibers could combine the structural and mechanical properties associated to the high molar mass of the polymer, and the bioactivity-driven applications promoted by the low molecular mass.

Wet spinning of chitosan represents an important research area, addressing the limitations of chitosan-based materials, particularly in enhancing their mechanical properties [73,74]. These properties are often diminished in the wet state due to the highly hydrophilic nature of chitosan. The objective of this study is to combine high molecular weight (HMW) chitosan solutions with low contents of CNFs and low molecular weight chitosan (LMW, “oligomers”), to develop chitosan-based spun composite fibers of high strength, by modulating the microstructure and properties of fibers in a wet-spinning process. These fibers have promising applications in biomedicine, for example, as biomedical textile fibers. The approach used in this work aims to enhance the performance of chitosan-based bioactive materials, with potential biomedical applications like in tissue engineering, suture threads, knitted fabrics, wound healing, and repairing and regeneration of mechanically demanding tissues.

## 2. Materials and Methods

### 2.1. Starting High Molecular Weight Chitosan and Its Depolymerization into Low Molecular Weight Chitosan

Chitosan (CHI) of high molecular weight (HMW) derived from squid pen chitin was supplied by Mahtani Company (Type: CHITOSAN 144, Batch No. 20120926, Veraval, Gujarat, India) The degree of acetylation (DA) was 2.5%. It was determined using ^1^H NMR spectroscopy, as described by Hirai et al. [62]. Chitosan (10 mg) was dissolved in 1 mL of deuterium oxide (D_2_O) that had been acidified with 5 μL of hydrochloric acid (HCl) (12 M). The experiment was carried out using a Bruker ALS 300 spectrometer (300 MHz) at 298 K (Bruker GmbH, Ettlingen, Germany).

Chitosan of low molecular weight (LMW) was obtained by depolymerization of the above HMW chitosan. The depolymerization was carried out by nitrous deamination with sodium nitrite (NaNO_2_), as described in previous works [29]. Chitosan at concentration of 4% (*w*/*v*) was dissolved in a stoichiometric amount of acetic acid to protonate the chitosan amine groups. Then, NaNO_2_ was added to obtain a given molar ratio *r* of glucosamine (GlcN) to NaNO_2_ as the following:

(1) $$r=\frac{n_{GlcN}}{n_{{NaNO}_{2}}}$$

The base solution of NaNO_2_ was added dropwise to each of the chitosan sample solutions while being stirred vigorously for 24 h at room temperature (RT), allowing the NaNO_2_ to react with the glucosamine units and depolymerize the chitosan chains at a given extension depending on the specifically used molar ratio *r*. Then, the chitosan was precipitated, centrifuged, washed and lyophilized. First, the precipitation of chitosan was carried out with sodium hydroxide (NaOH) at pH 9. Second, the centrifugation was realized at 1000 rpm for 10 min at 20 °C. While the supernatant was carefully poured into a beaker to avoid loss of coagulated chitosan, deionized water was added to the centrifugation vessels to wash the precipitated chitosan. The pH of the discarded liquid was measured to confirm that the CHI was completely washed. Finally, the freeze-drying of the depolymerized sample was performed.

### 2.2. Cellulose Nanofibers (CNFs)

Gel-like suspensions of nanofibrillated cellulose (CNF) were created at the Centre Technique du Papier (CTP, Grenoble, France) from bleached pine sulfite dissolving pulp using a mechano-enzymatic process developed from Pääkkö et al. [63]. The pulp was refined at 4.5% consistency for 25 min with a 12″ single disk refiner before 1 h incubation at 50 °C with a solution of endoglucanase FiberCare R^®^ (Novozymes Biologicals, Paris, France) at pH 5.0. The digested samples were processed further to generate a pulp solution with an SR (Schopper-Riegler) value more than 80 and a mean fiber length less than 300 µm. An Ariete homogenizer was used to process 2% (*w*/*w*) fiber suspensions, with one step at 1000 bar followed by three steps at 1500 bar. The surface charge density of the obtained CNFs was 40–80 mmol/kg. In other words, they had carboxylate moieties that were weakly charged. As previously reported, atomic force microscopy (AFM) was used to characterize the cellulose nanofiber morphology, which revealed an entangled network of linked nanofibrils with an average width of 35.2 ± 8.1 nm and bundles up to 100 nm wide.

### 2.3. Size Exclusion Chromatography Coupled to Multi-Angle Laser Light Scattering (SEC/MALLS). Characterization of Chitosan Molecular Weight

The chitosan molecular weight was measured using size exclusion chromatography (SEC) and multi-angle laser light scattering (MALLS) [73]. To prepare the eluent, a 0.1% (*w*/*v*) chitosan solution was prepared in an acetic acid/ammonium acetate buffer with a pH of 4.5 (AcOH (0.2 M)/AcONH4 (0.15 M). Prior to SEC measurements, the fluid was filtered via 0.22 µm pore size membranes (Millipore, Burlington, MA, USA). The chromatographic apparatus consisted of an IsoChrom. The LC pump (Spectra-Physics, Charbonnières les Bains, France) was linked to a Protein Pack 200 SW column (WATERS, Saint-Quentin-en-Yvelines, France) and a TSK gel G6000 PWXL column (Merck, Saint-Quentin-Fallavier, France). A Dawn DSP (Wyatt Technology, Toulouse, France) Multi-Angle Laser Light Scattering (MALLS) detector at 632.8 nm was connected to a WATERS 410 differential refractometer in Saint-Quentin-en-Yvelines, France.

### 2.4. Preparation of Viscous Collodions Containing Low- (LMW) and High-Molecular Weight (HMW) Chitosans with Cellulose Nanofibrils

To spin fibers, viscous formulations with low and high molecular weight chitosans (LMW and HMW) and with dispersed cellulose nanofibers of the type nanofibrillated cellulose (CNF) as described above, were prepared to obtain compositions as displayed in Table 1. A fine powder of high molecular weight (HMW) chitosan was mixed with different percentages of low molecular weight (LMW) chitosan (produced for molar ratio *r* of glucosamine (GlcN) to NaNO_2_ of 20 and 50) to yield mixed chitosan acetate solutions (hereafter named collodions or dopes) containing a total chitosan concentration of 4% (*w*/*w*) (Table 1). The aqueous dispersions were sonicated with a SONOPULS ultrasonic homogenizer (Bandelin electronic GmbH, Berlin, Germany) with a Bandelin titanium alloy ultrasonic probe Booster Horn SH 213G of 13 mm diameter, for 5 min at 40% amplitude. The acetic acid was added in stoichiometric amounts to protonate the amine moieties of chitosan (DA = 2.5%) to solubilize the chitosan. Finally, the mixture was kept under mechanical stirring overnight.

### 2.5. Rheological Analysis of of Cellulose Nanofiber-Filled High/Low Molecular Weight Chitosan Viscous Formulations

An AR2000 rheometer (TA Instruments Ltd., New Castle, DE, USA) with a cone-plate geometry (diameter: 25 mm; angle: 4°) was used to assess the rheological properties of various chitosan-based viscous formulations at 25 °C. The gap size was 0.116 mm, and a solvent trap was employed to prevent drying or evaporation. The cone-plate design ensures a consistent shear rate throughout the sample. The analysis was conducted in triplicate, operating in continuous mode within a shear rate range of 0.01 to 500 s^−1^. Flow curves, which are plots of steady-state shear viscosity against shear rate for the viscous collodions, were generated to determine the Newtonian viscosity in the low shear rate range plateau.

### 2.6. Gel Spinning of Chitosan Mixture Collodions

For the processing of the fibers, a gel spinning setup was used which consisted of a 30mL syringe. The syringe contained the chitosan/cellulose nanofibers viscous formulation, which was connected to a controlled air-pressure clip of a Performus I Nordson EFD dispenser linked to a compressed air source [33,75,76,77]. The syringe was attached to a conic needle with a tip diameter of 0.58 mm (gauge 20, pink, Nordson EDF). Briefly, the biopolymer viscous extrudate was directed into a coagulation bath containing 3M NaOH. The coagulated hydrogel macrofilament was pulled with aid of a first direct current electrical motor (DC motor). Afterward, the hydrogel macrofiber passed through a washing bath of deionized water, stretched and pulled by means of a second spinning DC motor. The third and fourth spinning DC motors were implemented to further stretch the fibers, while allowing them to dry. Finally, the spun fibers were collected onto a bobbin. Composite fibers of chitosan/cellulose nanofibers were processed using viscous formulations with a total chitosan concentration (LMW plus HMW) of 4 wt%, and CNF contents of 0.3, 0.4, and 0.5 wt%, respectively.

### 2.7. Fourier-Transform Infrared (FTIR)

The chitosan/cellulose nanofiber spun fibers were characterized by FTIR in attenuated total reflection (ATR) mode using an FTIR-ATR 65 spectrometer (PerkinElmer). Spectra in the range of 400–4000 cm^−1^ were recorded. Reference spectra of the used chitosan powder and of the nanofibrillated cellulose (CNF) were also recorded.

### 2.8. Scanning Electron Microscopy (SEM)

The fracture and the lateral surfaces of the chitosan/cellulose nanofiber spun fibers were observed using a FEI Scios DualBeam FIB/SEM microscope at an accelerated voltage of 5 kV after sputter-coating an ultrathin Au layer.

### 2.9. Transmission Electron Microscopy (TEM) of Ultrathin Fiber Section

Spun fibers were fixed in 10% formalin, 2% glutaraldehyde in Dulbecco’s phosphate buffered saline (DPBS) overnight at 4 °C. The sample was then incubated in 1% uranyl acetate in 70% ethyl alcohol overnight at 4 °C and further dehydrated in increasing concentrations of ethyl alcohol and finally acetone. After embedding in Durcupan resin, ultrathin sections containing the spun fiber were cut either parallel to the fiber axis (lateral view), or perpendicular (cross-section view) by using a UC7 Ultramicrotome (Leica, Wetzlar, Germany), and collected on Formvar-coated copper grids. Post staining was done for 1 min with 3% lead citrate followed by imaging using a Zeiss Leo 912 (ZEISS, Oberkochen, Germany) transmission electron microscope operating at 80 kV.

### 2.10. X-Ray Scattering (WAXS)

X-ray scattering analyses at wide angles (WAXS) of the collected spun fibers were performed by using synchrotron and laboratory X-ray sources. The synchrotron X-ray analyses were performed at the D2AM/BM2 beamline at the European synchrotron radiation facility ESRF (Grenoble, France). The processed fibers were wrapped ten times around a metal sample holder with a hollow center to allow the X-ray beam to pass through a bundle of fibers as previously described [33]. The setup allowed for X-ray scattering and diffraction analyses in transmission mode. Synchrotron wide angle X-ray scattering data were collected at a wavelength λ = 0.77 Å using a 2D detector WOS (IMXPAD company, La Ciotat, France). Chromium oxide was used as standard to calibrate the scattering vector q-range. In the data treatment, transmission corrections and background subtraction were performed. The WAXS analyses with laboratory source were also recorded in transmission mode in a Gemini A Ultra diffractometer (Agilent Technologies XRD UK), with an Atlas CCD detector and using the copper K-alpha radiation.

### 2.11. Thermogravimetric Analysis (TGA)

Thermogravimetric analysis of the CHI/CNF spun fibers was performed on a STA449 F5 Netzsch thermal gravimetric analyzer. Approximatively 10 mg of cut fibers were weighed in a platinum pan and heated from room temperature (~25 °C) up to 650 °C at a heating rate of 10 °C/min, under nitrogen atmosphere with a flow rate of 100 mL/min.

### 2.12. Tensile Testing

Microtensile tests of the chitosan/cellulose nanofiber spun fibers were performed using a DEBEN mini-tester equipped with a 20 N load cell. All tests were carried out at room temperature, and a constant strain rate of 0.5 mm/min. Fiber segments were fixed to the tester allowing a span length of 3 mm between the clamps, corresponding to the initial length (L_0_) before straining of the fiber. The nominal stress σ was calculated as the ratio of the applied force F to the initial cross-sectional area A of the fiber (σ = F/A), whose dimension was determined using a light optical microscope (Olympus BX Series, Hamburg, Germany). The nominal strain ε was expressed as the ratio of the extension of the fiber with respect to its initial length L_0_ (ε = ΔL/L_0_ = (L − L_0_)/L_0_). The Young’s modulus (E), yield stress (σ_y_) and strain (ε_y_), ultimate stress (σ_b_), and strain at break (ε_b_) were determined from the obtained stress–strain curves, considering at least ten replicates (n = 10) for each spun fiber formulation.

## 3. Results and Discussion

### 3.1. Rheological Behavior of Mixture Solutions of Low (LMW) and High-Molecular Weight (HMW) Chitosan Viscous Formulations Filled with Cellulose Nanofibers (CNF)

**Figure 1:**
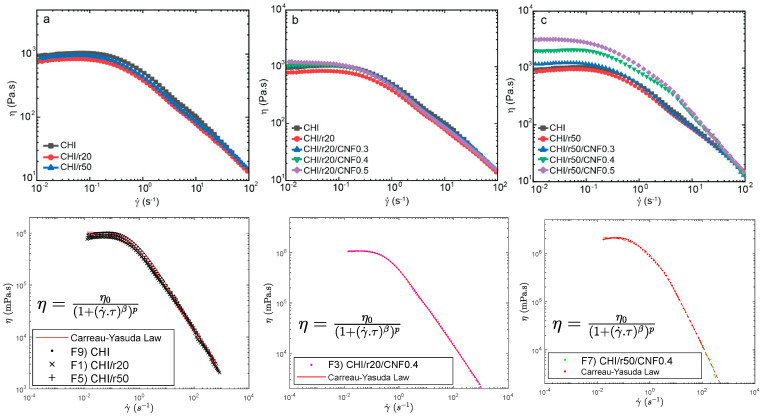
Flow diagrams showing the evolution of the viscosity (*η*) vs. shear rate ($\dot{\gamma }$) of: (**a**) HMW chitosan with LMW chitosan (“oligomers”) produced for ratio r = 20 or r = 50 (formulations: F1 or F5; Table 2, and CHI (HMW) reference solution (formulation: F9; Table 2), (**b**) CNF-filled CHI/LMWr20 suspensions (formulations: F2, F3, F4; Table 2) and CHI reference solution, and (**c**) CNF-filled CHI/LMWr50 suspensions (formulations: F6, F7, F8; Table 2) and CHI reference solution. (***Bottom***) Examples of modeling of the flow diagrams with Carreau-Yasuda law (Equation (2)).

Figure 1 presents the flow diagrams of the different formulations. These include chitosan/cellulose nanofiber mixtures prepared with low molecular weight chitosan, which were synthesized at molar ratios (*r*) of glucosamine to NaNO_2_ equal to 20 and 50. As expected, adding low molecular weight to the system, initially consisting of high molecular weight, reduced the Newtonian viscosity of the collodions. The HMW chitosan will have a reduced polymer movement, leading to a higher viscosity compared to LMW chitosan [40]. Thus, chitosan concentration and average molecular weight of the mixture allow for control over viscosity. Then, the incorporation of CNF into the mixture of high and low molecular weight chitosan re-increased the zero-shear viscosity. This effect was enhanced when adding the chitosan of intermediate low molecular weight. This is specially enhanced when adding “higher” low molecular weight (Mw) as for r50 to the systems having the highest contents of CNF (0.4 and 0.5% (*w*/*w*)). This might be explained due to strong interactions between CNF and chitosan macromolecules in those suspensions of higher concentrations of CNF and higher average molecular weight of chitosan [33]. Figure 1 illustrates the relationship between viscosity (*η*) and shear rate ($\dot{\gamma }$) for suspensions containing high (HMW) and low molecular weight chitosan (LMW for molar ratios r20 and r50), reinforced with varying CNF contents (0, 0.3, 0.4, and 0.5 (*w*/*w*)). Each dispersion exhibits a nonlinear, non-Newtonian shear-thinning behavior typical of CHI solutions, where η decreases as $\dot{\gamma }$ increases due to the disentanglement of polymer chains and the disruption of the initial polymer solution structure, which enhances chain orientation [19,32,34]. Further comparisons were made for the systems of CHI/r20/CNF and CHI/r50/CNF at different CNF contents (Figure 1b,c). The use of low molecular weight produced for r50 introduces longer chains than those produced for r20. Together with CNFs, they formed intermolecular aggregates that limited polymer chain movement and disentanglement, resulting in a more rigid structure [40], also increasing shearing effects.

At higher shear rates, the typical non-Newtonian shear thinning behavior of chitosan solutions was observed, with a Power law decrease of viscosity with increasing shear rates [34]. The flow behavior is related to the disentanglement of the chitosan chains, also inducing chain orientation. Overall, flow-induced orientation tends to form an anisotropic structure under the action of a shear field [19,32]. In the highest shear range, the viscosity similarly decreased in the “pure” chitosan solutions and in the chitosan/cellulose nanofiber viscous suspensions, revealing that the shear-thinning behavior in the latter is governed by the disentanglement of chitosan chains and practically not affected by the presence of the nanofibers, which should become oriented in the flow direction [34,78]. This is advantageous to achieve the extrusion of nanofiber reinforced viscous chitosan systems without compromising the extrudability of chitosan systems themselves as in fiber spinning processes. This should be the first premise to achieve the processing of nanofiber-filled chitosan fibers by wet spinning to develop fibers of improved mechanical performance, as envisaged in this work.

A Carreau-Yasuda law (Equation (2)) was used to model the flow diagrams (*η* vs. shear rate) of the chitosan solutions [79,80,81,82] and the chitosan/cellulose nanofiber viscous suspensions that also contained low molecular weight chitosan as illustrated in Figure 1 (*Bottom*) as the following:

(2) $$\eta =\frac{\eta_{0}}{{\left(1+{\left(\dot{\gamma }\cdot \tau \right)}^{\beta }\right)}^{p}}$$

where *η*_0_ is the Newtonian or zero-shear viscosity, *τ* is the transition time, *β* ist the exponent that accounts for the width of the transition region between the zero-shear viscosity and the Power law region, and *p* = (1 − *β*)/*β* is the viscous exponent. The parameters obtained for the different formulations after modelling with the Carreau-Yasuda law are displayed in Table 2.

For the CNF 0.4 and 0.5% (*w*/*w*), having low molecular weight chitosan from used ratio r50, a second fast flow mode was introduced to obtain a good fit with the model. This could be explained to a representative interaction between the CNF and the low molecular weight chitosan of intermediate Mw (for r50) specially being significant at those high contents of cellulose nanofibers (0.4 and 0.5% (*w*/*w*)).

### 3.2. Structure of Spun Fibers. Composite Fibers and Relation to Macromolecular Structure of Chitosan Determined by SEC-MALLS

**Figure 2:**
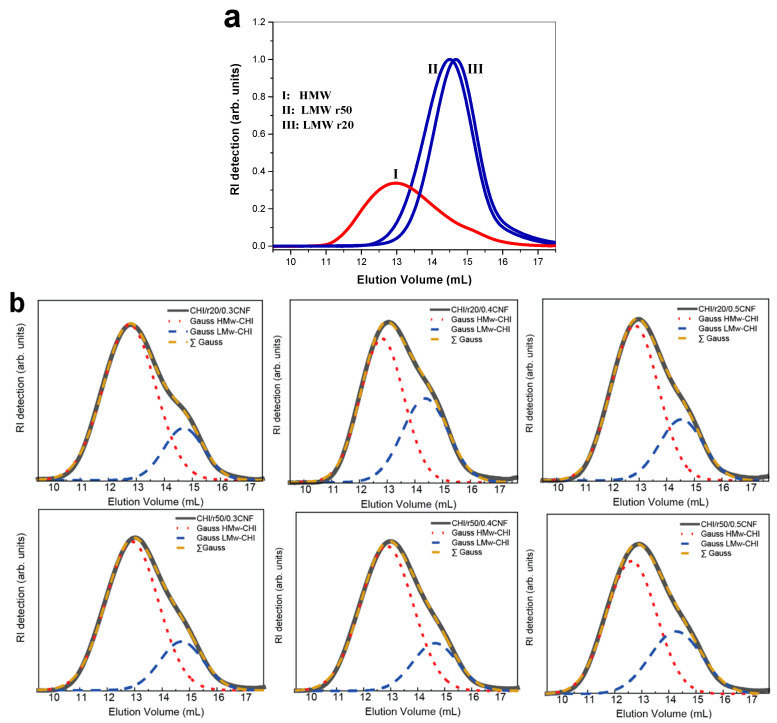
The SEC/MALLS analysis of the colloidal dispersions for: (**a**) low (LMW, blue) and high molecular weight (HMW, red) chitosan weight distribution, and (**b**) chitosan molecular weight distribution in the processed spun fibers containing low (LMW) and high molecular weight (HMW) chitosan. The curves in (**b**) are deconvoluted using the two Gaussian (Gauss HMw-CHI for HMW chitosan (red), and Gauss LMw-CHI for LMW chitosan (blue)), with a sum (ΣGauss) that coincides with the experimental curve of the SEC chromatogram.

Figure 2a displays the size-exclusion chromatograms (SEC) of the starting chitosan of high molecular weight (HMW) as well as of the low molecular weight chitosan (LMW) produced by nitrous deamination depolymerization of the HMW sample. Figure 2 confirmed the decreasing of chitosan molecular weight by varying the molar ratio r of glucosamine to NaNO_2_ (for example: r20 and r50). Figure 2 also shows the chromatograms of the chitosan contained in the different fiber formulations. For this analysis, the spun fibers were redissolved in buffer pH 4.5 (AcOH (0.2 M)/AcONH4 (0.15 M) for solubilization of chitosan and the obtained system was subsequently filtered through 0.22 µm pore size membranes (Millipore). The SEC analysis allowed verifying the obtaining of bimodal distribution of molecular weights, containing chitosan of high and low molecular weight after the fiber spinning process, with elution volumes corresponding to that of the HMW and LMW chitosan used in viscous collodion. Due to the non-solubility of CNF in buffer pH 4.5, it was possible to reconstruct the chitosan weight distribution within the fiber composite. Figure 2b shows, for each formulation, two Gaussian curves representing the distribution of components of HMW and LMW chitosan, respectively. It confirmed that despite the high mobility of the low molecular weight chains, they were not removed during coagulation in base or water washing baths and remain present in the final fibers (Figure 2b). Table 3 summarizes the obtained molecular weight and polydispersity index (Đ) of chitosan in the different samples.

Table 3 shows the molecular weight and polydispersity index (Đ) of the different chitosan samples. The samples containing pure chitosan of high molecular weight HMW (CHI) or of low molecular weight LMW (produced for ratios r20 and r50) exhibits a relatively low Đ (≤1.6), indicating a narrow polymer molecular weight distribution. It is important to note that the degree of acetylation (DA) in CHI remains unchanged after the depolymerization reactions by nitrous deamination, suggesting that the acetylation process is not affected by the changes in molecular weight during this reaction.

### 3.3. Fourier-Transform Infrared Spectroscopy (FTIR)

**Figure 3:**
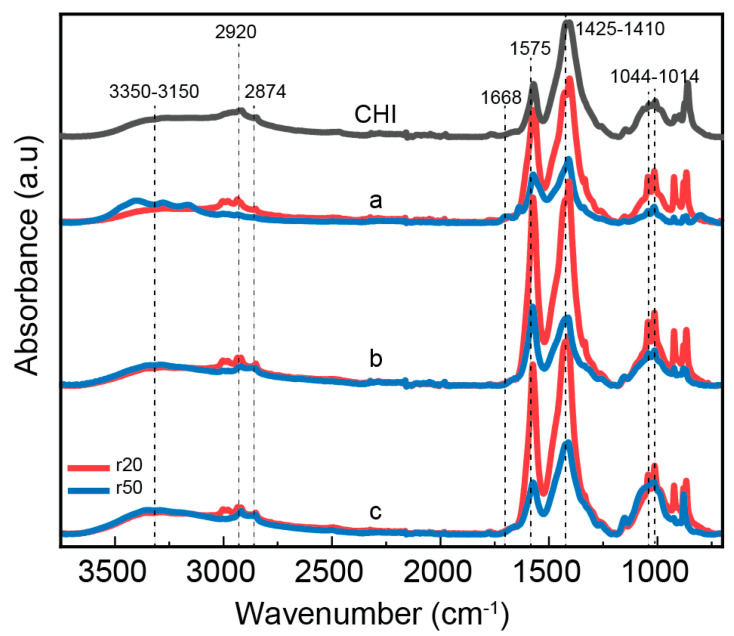
ATR-FTIR spectra of spun composite fibers consisting of high molecular weight chitosan (HMW), low molecular weight chitosan (LMW) as obtained using different molar ratios of glucosamine to NaNO_2_ (r20: red; r50: blue), and cellulose nanofibers (CNF) at different contents: (a) 0.3; (b) 0.4 and (c) 0.5%; as well as CHI) the reference spectrum of the pure high molecular weight chitosan fibers (HMW).

The bonding mechanism between chitosan and CNF in the composite fibers was investigated using the Fourier transform infrared spectroscopy (Figure 3). Both chitosan alone and chitosan/CNF composite fibers exhibit a broad absorption band between 3350–3150 cm^−1^, corresponding to O-H and N-H stretching vibrations. This indicates the formation of intermolecular hydrogen bonds between CNFs and CHI molecules [50]. The peaks at 2934 and 2852 cm^−1^ are attributed to the asymmetric and symmetric stretching vibrations of the C-H group, respectively [52]. The carbonyl band, which is usually observed at ∼1670 cm^−1^ for chitosan in its free amine form, slightly shifted to a lower wavenumber when acetic acid was added to chitosan to form a chitosan-acetate salt complex as it can persist in our fiber processing. Indeed, for some spun fibers the presence of remaining sodium acetate was still revealed after washed as observed in the X ray diffraction patterns (see below). A characteristic absorption related to the amide I group in CHI was observed as a shoulder at 1671 cm^−1^, corresponding to C=O stretching [53,54]. Additionally, the amide II group presents a peak at 1574 cm^−1^, which corresponds to both N-H bending vibration and NH^3+^ symmetric deformation. An increase in CNF content leads to a rise in the intensity of the latter peak, indicating favorable interactions (electrostatic and hydrogen bonding) between the COO- group of cellulose and the amine group of CHI [52,55]. In summary, the band region 1630–1540 cm^−1^ is normally assigned to the amide group, which suggests the interaction of amino groups of chitosan with carboxyl groups as expected in fibers of chitosan-acetate interacting with partially carboxylated cellulose nanofibers. Furthermore, the methyl distortion is observed approximately at 1380 cm^−1^, and the stretching of the C–O groups from primary and secondary hydroxyl groups shows characteristic bands at 1044 and 1013 cm^−1^, respectively [55,56]. Finally, CHI and other polysaccharides exhibit a fingerprint region with peaks around 800–1000 cm^−1^.

### 3.4. Morphology and Microstructure of Fiber Yarns

**Figure 4:**
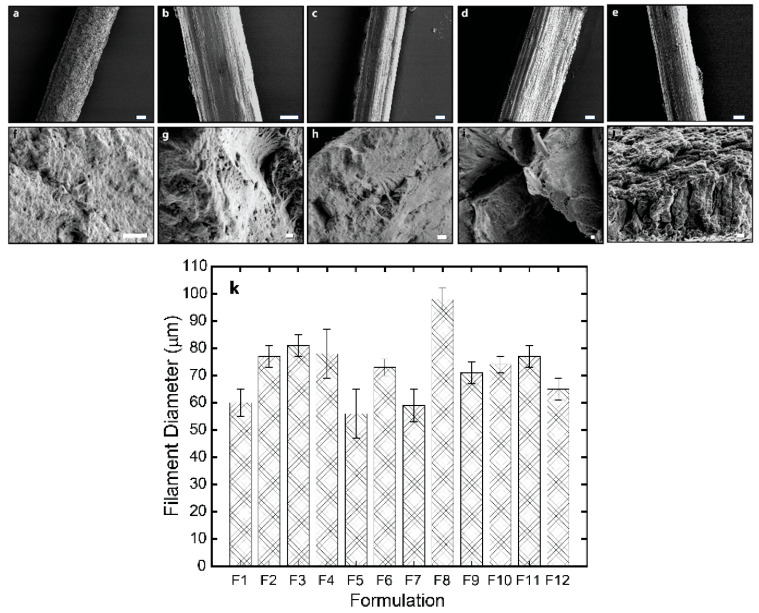
The SEM micrographs of the lateral (top) and fracture cross-section (bottom) surface of the spun HMW/LMW/CNF composite fibers of (**a**,**f**) CHI/r20/CNF0.3; (**b**,**g**) CHI/r20/CNF0.4; (**c**,**h**) CHI/r50/CNF0.4; (**d**,**i**) CHI/r20/CNF0.5; and (**e**,**j**) CHI/r50/CNF0.5. The scale bars for the lateral surface are 20 µm, and the scale bars for the fracture cross-section surface are 2 µm. (**k**) The histogram showing the mean fiber diameter obtained with the different HMW/LMW/CNF collodion formulations (F1–F12, see Table 1).

The lateral and cross-sectional surface morphology of the spun fibers was analyzed using scanning electron microscopy (SEM), and transmission electron microscopy of ultrathin sections, which analyses were performed transversally (cross-section) or along the axial direction of fibers. The SEM micrographs in Figure 4 reveal the good compatibility of cellulose nanofibers with chitosan matrix in composites, due to the similar structures of the polysaccharides chitosan and cellulose as well as the possibility of electrostatic interaction between matrix and nanofibers, especially at the surface of the cellulose nanofibers. The spun fibers display a relatively homogeneous inner microstructure, which confirms strong compatibility between nanofiber filler and matrix and thereby an effective formation of composite, with the presence of also low molecular weight chitosan into the matrix, and low contents of CNF [33]. For the higher CNF contents (0.4, and 0.5% (*w*/*w*)), the outer surface of fibers is characterized by a fibrillar topography for formulations containing any of the synthesized low molecular weight chitosan (for r20 and for r50), which effect should result from the spinning and stretching process and especially enhanced for higher nanofiber contents in composites. The orientation of that outer fibrillar microstructure observed by SEM aligns parallel to the stretching direction [15]. In the cross-sectional microstructure, an internal homogeneity is also observed for all spun fibers with CNF contents 0.3 and 0.4% (*w*/*w*) as well as for the fibers with CNF content of 0.5% with the low molecular weight chitosan of lower Mw (for r20). In contrast, for spun fibers with that higher CNF content of 0.5% incorporating low molecular weight chitosan of higher Mw (for r50) it seems that the homogeneous microstructure starts to be disrupted, due to the higher concentration of nanofibers and increase of average molecular mass of chitosan chains [33]. Figure 4k shows that the average diameter of the spun fibers ranged from 60 to 98 µm, with the presence of r50 “oligomers” and highest CNF content (0.5% (*w*/*w*)) contributing to an increase in diameter.

**Figure 5:**
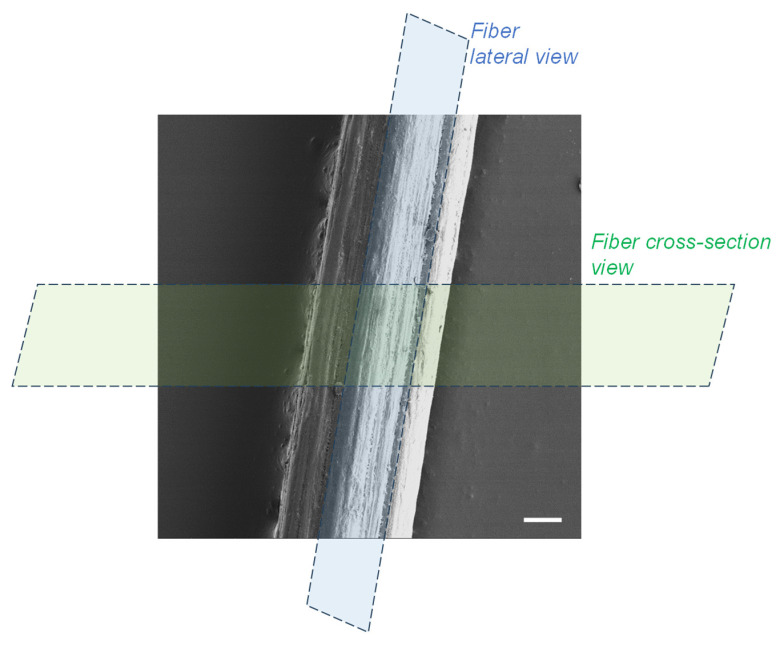

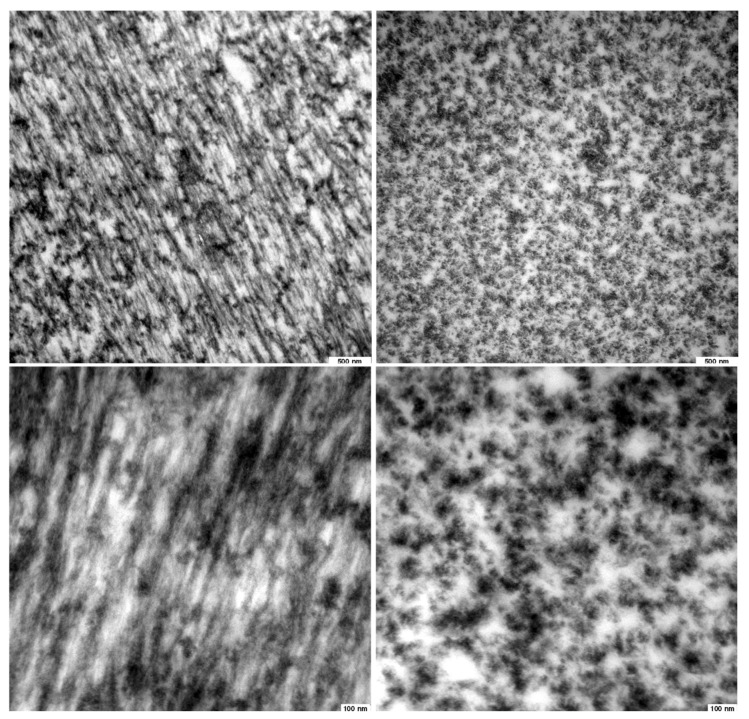
Micrographs of ultrathin section of spun hybrid fiber produced from formulation F7 (CHI/r50/CNF0.4), which consists of low molecular weight chitosan (for r50) at 0.6% (*w*/*w*), cellulose nanofiber content 0.4% (*w*/*w*), and high molecular weight chitosan at 3.4% (*w*/*w*). (***Top***) The SEM micrograph of fiber overview, with the scale bar of 20 μm. (***Middle-left***) The TEM micrograph of fiber cut parallel to the fiber axis (Lateral view). (***Middle-right***) The TEM of fiber cut perpendicular to the fiber axis (Cross-section view). (***Bottom-left***) Higher magnification photo of fiber cut parallel to the fiber axis (Lateral view). (***Bottom-right***) Higher magnification photo of fiber cut perpendicular to the fiber axis (Cross-section view).

Microscopy observations at higher resolution, which were performed by transmission electron microscopy (TEM) of ultrathin fiber sections, allowed better characterizing the inner microstructure of the fibers. In the micrographs of Figure 5, a pattern of parallel arrangements with a period width of 50–100 nm is observed when looking at sections cut along the spun fiber axis (Figure 5 *Middle*). This observed period in the patterns is in the range of the average width of the nanofibrils constituting the nanofibrillated cellulose filler CNF, which nanofibrils have an average width of 35.2 ± 8.1 nm with bundles up to 100 nm (see Section 2.2 Cellulose Nanofibers). The TEM results might reveal the good dispersion of the cellulose nanofibers in the chitosan matrix, even at the resolution of single nanofibril bundles as present in the starting cellulose nanofiber material (CNF suspension), which was added to the chitosan-based viscous collodion to be spun into hybrid fibers. A preferential orientation in the direction of the fiber axis was observed in the patterns, which might coincide with the orientation of cellulose nanofibers inside the composite, and/or the orientation of the semicrystalline chitosan produced after stretching and drying while spinning [33]. The cross-sectional ultrathin section of the spun fibers in the TEM micrographs (Figure 5 *Bottom*) shows homogeneously distributed bright and dark regions, which might correspond to regions containing cellulose nanofiber and chitosan, respectively, matching with the patterns observed when looking at orientation along fiber direction as described above (Figure 5 *Middle*).

### 3.5. Crystalline Microstructure of Spun Fibers at Wide-Angle X-Ray Scattering (WAXS)

**Figure 6:**
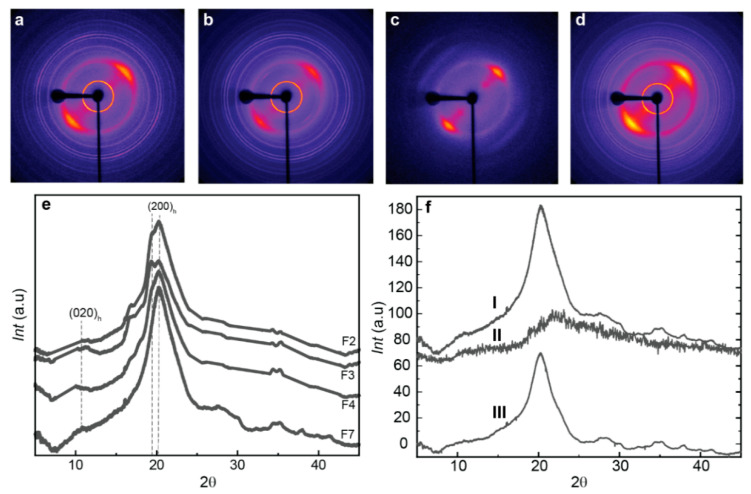
(**Top**) Two-dimensional wide-angle X-ray scattering (2D-WAXS) images of the spun CHI/OLIG/CNF fibers of (**a**) CHI/r20/CNF0.3, (**b**) CHI/r20/CNF0.4, (**c**) CHI/r50/CNF0.4, and (**d**) CHI/r20/CNF0.5 with fiber axis tilted by −45° from the vertical position. (***Bottom***) (**e**) Radial average over the 360° azimuth of the above 2D-WAXS images of spun fibers of varied CHI/OLIG/CNF formulations. (**f**) Procedures for the calculation of crystallinity indexes from WAXS diffraction patterns of the fibers. (**fI**) Diffraction diagram of the original CHI/r50/CNF0.4 fiber. (**fII**) Diffraction diagram of the amorphous chitosan sample. (**fIII**) Estimated crystalline contribution of CHI/r50/CNF0.4 fiber after subtraction of the area of the amorphous diffractogram II.

The hybrid chitosan/cellulose nanofiber spun fiber composites were examined using X-ray diffraction, which shows appropriate anisotropic behavior along the direction of fiber stretching (Figure 6), with preferential orientation of the hydrated chitosan allomorph as displayed in the 2D diffraction images by the arc signal of the corresponding crystallographic planes [83]. Figure 6e displays the X-ray scattering radial average profile curves with the main crystallographic peaks of the biopolymers. These peaks were observed at 2θ = 10.61° for the (020)_h_ planes of the hydrated crystalline polymorph of chitosan, and at 19.85° and 20.38° for the reflections of the (200)_h_ and (220)_h_ planes of the hydrated polymorph. The overall spectra are characteristic of hydrated CHI, like those described by Clark and Smith [42,49]. In some samples, the washing with water did not effectively remove the salt sodium acetate produced as byproduct during neutralization, and crystallographic signal of that salt was observed in the WAXS pattern of some fiber samples. For quantitative analyses of WAXS data, the sharp peak signals of salt were removed in the WAXS radial profile, to better investigate the crystalline aspects related to chitosan and cellulose.

**Figure 7:**
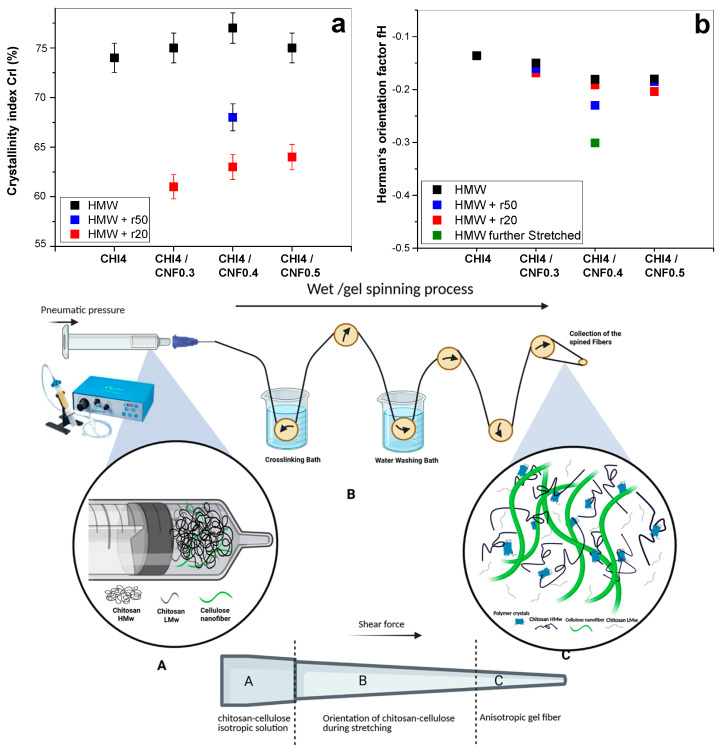
(**a**) The crystallinity index (CrI) and (**b**) the Herman’s orientation factor (fH) values of spun fibers obtained for different formulations containing high (HMW) and low molecular chitosan (LMW, with used molar ratio of glucosamine to NaNO_2_ of: r20 or r50), and cellulose nanofibers (CNF). (***Bottom***) Schematics summarizing the transition from isotropic collodions to anisotropic spun fibers, involving formation and orientation of crystalline materials due to shearing and molecular mobility during extrusion, stretching and drying in spinning of chitosan/cellulose nanofiber hybrid fibers.

Chitosan is a semicrystalline polymer, composed of both amorphous and crystalline phases [13]. The crystallinity index (CrI) gives insight into the percentage of crystalline regions with respect to the sum of amorphous and crystalline phases. Moreover, higher crystallinity typically leads to increased rigidity and thereby stiffness and strength of the material. To calculate the crystallinity index (CrI), we used the diffractogram of an experimentally obtained amorphous chitosan. The amorphous diffractogram was adjusted by a coefficient, to match the amorphous background in the diffractograms obtained for the spun fibers (Figure 6f). The CrI was determined by comparing the amorphous contribution with the total area of the diffractogram. Figure 7 presents the CrI values obtained for spun fibers of different formulations. It can be observed that the combined addition of LMW chitosan and CNF to HMW chitosan contributes to increasing the crystallinity in the fibers, with a higher contribution being observed when adding low molecular weight produced for r50 than for r20. This is a higher average molecular mass of chitosan will contribute to obtaining fibers of higher crystallinity. The observed increase in CrI is primarily associated with the presence of CNF, as chitosan polymer chains adsorb to the CNF surface during the coagulation and stretching processes, acting the nanofiber as nucleating points to promote the growth of chitosan crystals [33].

The Herman orientation factor (fH) can be calculated from the 2D WAXS images. The Herman equation (Equation (3)) defines and relates the orientation factor in relation to the crystallites within the fibers. To this end, the 2D wide angle X-ray scattering (WAXS) images were azimuthally sectorized, and the intensity peak around the (*h*,*k*,*l*) crystallographic peak was deconvoluted for each sector centered at the azimuthal angle *φ*_*h*,*k*,*l*_. For the calculation of the Herman orientation factor (fH), the average of the cosine squared of azimuthal angle *φ*_*h*,*k*,*l*._ is determined by integrating the measured peak intensity at each angle and dividing by the total integrated intensity [51] as the following (Equation (3)):

(3) $${\;\;f}_{h,k,l}=\;\frac{1}{2}3\langle \cos^{2}\varphi_{h,k,l}\rangle -\frac{1}{2}$$

The Herman’s factor values closer to −0.50 indicate a higher orientation of the crystals within the fibers, while values closer to 0 signify isotropic behavior. Interestingly, the addition of both chitosan “oligomers” and cellulose nanofibers contribute to enhance orientation (Figure 7). The Herman’s orientation factor values around −0.20 were achieved, with further enhanced orientation values of −0.23 for the formulation F7 (CHI/r50/CNF0.4), consisting of low molecular weight chitosan of intermediate Mw, of cellulose nanofiber content 0.4% (*w*/*w*), and of high molecular weight chitosan. Then, a further stretching of this fiber led to further increase alignment within the fibers and Herman’s orientation factor of −0.3 was achieved (Figure 7).

To summarize, the schematics in Figure 7 illustrate how the contribution of cellulose nanofibers, serving as nano-reinforcement and surface for nucleation of chitosan crystallites, contributes to the increase crystallinity and orientation. This effect combined with the shearing during extrusion, the stretching and drying during spinning, yields fiber composites of enhanced crystallite orientation. This is specially enhanced when incorporating into the CHI/CNF formulation a chitosan of intermediate low molecular weight chitosan like Mw: 4.4 × 10^4^ g/mol produced for r50. The intermediate polymer chain lengthes were produced through the depolymerization (by nitrous deamination) by using a molar ratio *r* of glucosamine to NaNO_2_ of r50, which yielded an intermediate molecular weight of chitosan of Mw of 4.4 × 10^4^ g/mol. This Mw was higher (i.e., intermediate) than the low molecular weight of Mw of 2.9 × 10^4^ g/mol obtained by using a molar ratio *r* of glucosamine to NaNO_2_ of r20. Thus, the effect of enhanced crystallinity and orientation could be attributed to the presence of shorter chains, but not as short as for r20 (Mw: 2.9 × 10^4^ g/mol), which ensures higher molecular mobility of shorter chains, but still allowing for some entanglement of the intermediate Mw chains (Mw: 4.4 × 10^4^ g/mol) to sense shearing and contribute to orientation (Figure 7). These interpretations could serve as the basis for the analysis of the spun fiber composite properties like thermal and mechanical in the following sections.

### 3.6. Thermal Properties

**Figure 8:**
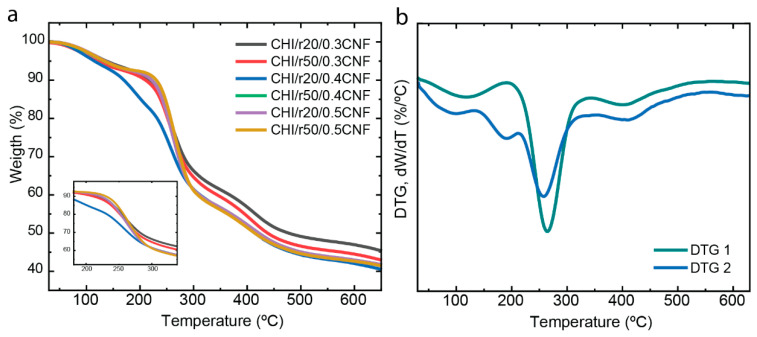
(**a**) The thermogravimetric analysis (TGA) and (**b**) derivative thermogravimetry (DTG).

The thermal stability and decomposition patterns of the composite fibers (CHI/LMW/CNF) were evaluated using thermogravimetric analysis (TGA). Figure 8 demonstrates how the presence of chitosan “oligomers” and nanometric CNF influences the thermal behavior of the CHI matrix. The use of CNF fibers is known to enhance the thermal stability of composite fibers [33,57,58]. Most of the samples exhibit similar decomposition patterns, with an initial mass reduction observed at around 100 °C, which corresponds to water desorption associated with the hydrogen bonding in the polysaccharide/water structure. The next phase, visible at 260 °C, corresponds to the decomposition of the polysaccharide macromolecules of both CHI and CNFs. Finally, at approximately 400 °C, the degradation of any residual sodium acetate from the neutralization step occurs, which was not entirely removed during the washing steps with water when spinning the composite fibers [33,59,60,61].

Interestingly, the CHI/r20/CNF0.4 formulation shows a different behavior compared to the other samples, with three distinct degradation steps after water evaporation (around 100 °C). The first degradation step, at 189 °C, is likely related to the breakdown of the “weak link” of CHI chains and added oligomers [49]. The subsequent degradation steps around 260 °C and 400 °C correspond to the decomposition of the macromolecules and the residual sodium acetate, respectively.

### 3.7. Micromechanical Properties

**Figure 9:**
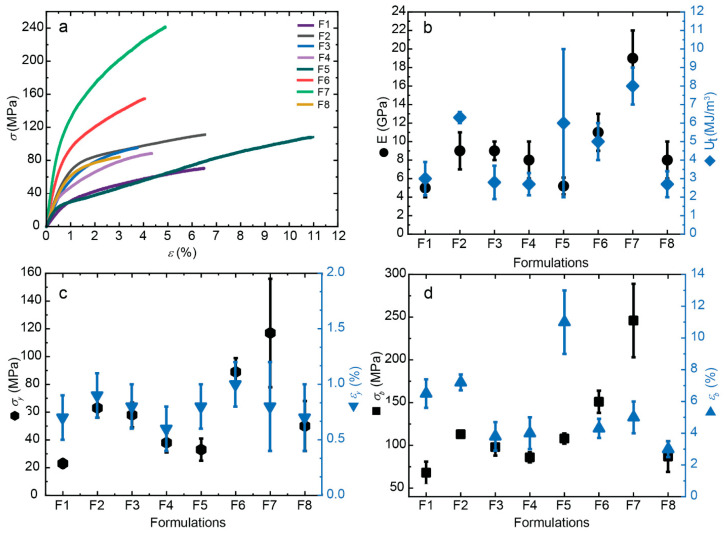
Tensile mechanical properties of spun CHI/LMW/CNF fibers obtained for different formulations. (**a**)The nominal stress–strain curves. Evolution of (**b**) Young’s modulus *E* and toughness *Ut*, (**c**) the stress (*σ*_y_) and strain (*ε*_y_) values at the yield point, and (**d**) the stress-at-break (*σ*_b_) and strain-at-break (*ε*_b_), with the increase of CNF content in spun fibers.

The incorporation of CNFs up to 0.4 wt% into the CHI matrix leads to a significant enhancement in the overall mechanical performance of the composite fibers. The addition of CNFs improves the nanocomposite’s mechanical properties, attributed to the high aspect ratio of the nanofiber reinforcement, which promotes efficient stress transfer between the matrix and the fibers [33]. This study focuses on examining the influence of “oligomers” on the mechanical behavior of the composite fibers, specifically evaluating parameters such as the Young’s modulus (E), yield point (σy and εy), stress-at-break (σb), elongation-at-break (εb), and toughness (Ut). Figure 9 illustrates the stress-strain curves for all formulations. When comparing composite fibers with different oligomers, r50 (F5–F8) demonstrates superior properties, like increased Young’s modulus E, yield stress σ_y_ and stress-at-break σ_b_, compared to those with r20 (F1–F4). Previous studies have shown that an optimal CNF content of 0.4 wt% yields the best results, which is why this condition was also selected for combination with the “oligomers” in this study. The oligomers significantly influence the stiffness (E) of the samples, with r50 samples reaching an average Young’s modulus value of 19 GPa, while with r20 a value of 9 GPa was reached, in both cases for a cellulose nanofiber content of 0.4 wt%. Furthermore, the yield and tensile strength (σ_y_ and σ_b_) of the composite fibers with r50 were as high as 101% and 151%, respectively. Specifically, for the toughness value Ut, some variability was observed with higher standard error observed in the sample formulation F5 with r50 low molecular weight chitosan, and without containing cellulose nanofibers. For the yield stress and stress at break, a large fluctuation of the values was also obtained for some composite fibers. The increase in the crystallinity index in fibers with LMW chitosan for r50 contributes to the highest stiffness of composites, whereas the shorter chains produced for r20 allowed for higher molecular mobility, leading to a reduction in overall mechanical performance.

To achieve the highest stiffness (E modulus) and strength (σ_y_) of spun fibers, in terms of chitosan molecular, the optimum composition seems to be that containing high molecular chitosan combined with a low molecular weight chitosan of not too short chains, this is, of intermediate Mw. The low molecular weight chitosan should have a Mw between that for r50 (Mw: 4.4 × 10^4^ g/mol) and that of HMW (Mw: 5.5 × 10^5^ g/mol). The results showed that both, low molecular weight chitosan and CNFs positively influence Young’s modulus and yield stress of spun hybrid fibers. However, only the chitosan molecular weight seems to have significant influence on fiber toughness. Thus, the highest Young’s modulus, yield stress and toughness are achieved at adding a low molecular weight chitosan of intermediate Mw value (e.g., for r50 sample) and cellulose nanofibers to the high molecular weight chitosan collodion, with the remark that the variation of CNF content in the considered CNF contents (0.3–0.5% (*w*/*w*)) is not significantly affecting toughness. To conclude, the best mechanical properties were achieved at combining low molecular weight from r50 with CNF at content 0.4% (*w*/*w*) with high molecular weight chitosan, when using collodions containing a total chitosan percentage of 4% (*w*/*w*). This composition also corresponds to the highest achieved values of crystallinity index (CrI) and the highest orientation within the fiber (see evolution Herman’s orientation factor fH)) as illustrated in Figure 7. It can be concluded that the crystallization, molecular mobility and shearing associated with the addition of low molecular weight chitosan of intermediate Mw, leads to an improvement of the fiber mechanical properties. It demonstrates the major role of introducing low molecular weight chitosan of intermediate Mw and cellulose nanofibers into chitosan collodions, to combine enhancement of crystallization and crystallites orientation in spinning process for the development of high strength fibers.

Tuning the molecular weight in chitosan formulations allows optimizing both the physico-chemical behavior and the physical properties for fiber processability and mechanical performance, as well as the biological properties through the incorporation of low molecular weight chitosan into high molecular weight-based systems, to advance biomedical and textile applications. The molecular weight of chitosan definitely should govern the physicochemical and biological properties as follows: (i) high molecular weight chitosan (HMW) contributes to increase viscosity in collodions, to lower the solubility into coagulation by approaching neutral pH, and it is better for structural applications like to produce films, hydrogels, fibers; and (ii) low molecular weight chitosan (LMW) contributes to higher chain mobility and solubility, to lower viscosity, and to better biological activity due to improved bioavailability. Especially shorter polymer chains, i.e., LMW chitosan, promote cellular uptake and interaction with biomolecules, conferring enhanced antimicrobial, antioxidant, anti-inflammatory, and immunomodulatory properties compared to HMW chitosan. Summarizing, a combination of HMW and LMW chitosans has a dual application domain. The HMW chitosan improves the engineering of high mechanical performance structural fibers, while the LMW chitosan promotes bioactivity-driven applications. This synergy of chitosan molecular weight combination, together to the incorporation of high strength cellulose nanofibers in spun composites, advances the achievement of functional materials for biomedical applications, as the development of biocompatible and biologically functional fibers of high mechanical performance.

## 4. Conclusions

Our results underscore the critical role of chitosan molecular weights and cellulose nanofibers in chitosan functional composite biomaterials. Chitosan-based fibers with increased stiffness and tensile strength were developed by incorporating small amounts of nanofibrillated cellulose (CNF) into a viscous chitosan (CHI) collodion solution of both high and low molecular weight chitosans in a gel spinning process. This approach resulted in anisotropic, nanofiber-reinforced biocomposite fibers with superior mechanical properties. Low molecular weight chitosan (LMW) allowed contributing to lower viscosity, higher chain mobility and solubility, while helping to enhance biological activity due to know improved bioavailability of shorter polymer chains. In contrast, high molecular weight chitosan (HMW) contributes to increase viscosity in collodions, lower the solubility by approaching neutral pH for gelation, and those larger polymer chains are better for structural applications like the processing of fibers, films, hydrogels. Then, the inclusion of CNFs in the CHI matrix plays a crucial role in shaping the morphological, thermal, and mechanical characteristics of the spun composite fibers. The CNFs significantly enhanced the stiffness, tensile strength, ductility, and toughness of the CHI-based fibers, owing to the remarkable mechanical properties, high aspect ratio, and strong compatibility of the cellulose nanofiber reinforcement with chitosan. This led to robust interfacial bonding between the components, involving both physical and covalent interactions.

Additionally, it was found that the mechanical and structural properties of these fibers are heavily influenced by the proportion of chitosan short chains (LMW) in the spinning solution. As the chitosan short-chain content increases, the viscosity of the collodion decreases, potentially facilitating the spinning process of collodions with higher polymer concentrations. Results from X-ray diffraction analysis show that the addition of LMW chitosan of intermediate Mw around 4.4 × 10^4^ g/mol produced the highest increase of the crystallinity index (CrI) and orientation in the fibers, yielding the best mechanical performance. The incorporation of low molecular weight chitosan oligomers, in combination with high molecular weight chitosan and cellulose nanofibers, allows the processing of fibers with tunable mechanical properties. While high molecular weight chitosan provides strength and stiffness through enhanced chain entanglement, the LMW chitosan improves ductility and extrudability of the collodions, leading to a synergistic enhancement in fiber performance. These findings underscore the critical role of polymer molecular weight tailoring in optimizing chitosan-based nanocomposite fibers for advanced biomedical and textile applications.

## Figures and Tables

**Table 1:** Summary of composition of formulations of low (LMW) and high molecular weight (HMW) chitosan filled with cellulose nanofibers (CNF), which were used in the fiber spinning.

Formulation		HMW	LMW		CNF
		% (*w*/*v*)	% (*w*/*v*)		% (*w*/*w*)
			r20	r50	
(F1)	CHI/r20	3.4	0.6	0	0
(F2)	CHI/r20/CNF0.3	3.4	0.6	0	0.3
(F3)	CHI/r20/CNF0.4	3.4	0.6	0	0.4
(F4)	CHI/r20/CNF0.5	3.4	0.6	0	0.5
(F5)	CHI/r50	3.4	0	0.6	0
(F6)	CHI/r50/CNF0.3	3.4	0	0.6	0.3
(F7)	CHI/r50/CNF0.4	3.4	0	0.6	0.4
(F8)	CHI/r50/CNF0.5	3.4	0	0.6	0.5
(F9)	CHI	4	0	0	0
(F10)	CHI/CNF0.3	4	0	0	0.3
(F11)	CHI/CNF0.4	4	0	0	0.4
(F12)	CHI/CNF0.5	4	0	0	0.5

**Table 2:** Flow parameters determined from the fitting of the viscosity vs. shear rate curves of different chitosan/cellulose nanofiber collodions also containing low molecular weight chitosan of used molar ratios r20 or r50 in the depolymerization reaction, by using the Carreau-Yasuda (Equation (2), Figure 1).

	Formulation	Log_10_ (*η*_0_/mPa·s)	*τ* (s)	*p*	*β*
(F1)	CHI/r20	5.9448	1.8582	0.7408	1.1043
(F2)	CHI/r20/0.3CNF	6.0558	2.3068	0.6299	1.2686
(F3)	CHI/r20/0.4CNF	6.0459	2.5849	0.5926	1.3280
(F4)	CHI/r20/0.5CNF	6.0878	2.9427	0.6281	1.2493
(F5)	CHI/r50	5.9867	2.0874	0.6140	1.2932
(F6)	CHI/r50/0.3CNF	6.0926	2.7594	0.4994	1.6075
(F7)	CHI/r50/0.4CNF	6.3558	0.8584	1.4199	0.8217
(F8)	CHI/r50/0.5CNF	6.5297	1.5515	1.2003	0.8862
(F9)	CHI	6.0229	1.4948	0.7200	1.1963

**Table 3:** Molecular weight and polydispersity index of CHI formulations.

	Formulation	Chitosan Mw (g/mol)	Chitosan Polydispersity Index Đ
(F1)	CHIr20	29340 (±0.63%)	1.3 (±1.33%)
(F2)	CHI/r20/0.3CNF	378100 (±0.91%)	2.8 (±1.35%)
(F3)	CHI/r20/0.4CNF	243600 (±0.62%)	2.3 (±1.02%)
(F4)	CHI/r20/0.5CNF	284100 (±0.69%)	2.4 (±0.91%)
(F5)	CHIr50	43840 (±0.49%)	1.5 (±0.89%)
(F6)	CHI/r50/0.3CNF	365500 (±0.98%)	2.8 (±1.37%)
(F7)	CHI/r50/0.4CNF	427100 (±1.03%)	2.7 (±1.65%)
(F8)	CHI/r50/0.5CNF	434600 (±1.04%)	2.7 (±1.79%)
(F9)	CHI	554000 (±1.06%)	1.6 (±1.59%)

## Data Availability

The original contributions presented in the study are included in the article, further inquiries can be directed to the corresponding author.
